# Essential oils of aromatic Egyptian plants repel nymphs of the tick *Ixodes ricinus* (Acari: Ixodidae)

**DOI:** 10.1007/s10493-017-0165-3

**Published:** 2017-09-01

**Authors:** Hesham R. El-Seedi, Muhammad Azeem, Nasr S. Khalil, Hanem H. Sakr, Shaden A. M. Khalifa, Khalijah Awang, Aamer Saeed, Mohamed A. Farag, Mohamed F. AlAjmi, Katinka Pålsson, Anna-Karin Borg-Karlson

**Affiliations:** 1 Division of Pharmacognosy, Department of Medicinal Chemistry, Biomedical Centre, Uppsala University, Box 574, 751 23 Uppsala, Sweden; 2 Department of Chemistry, Faculty of Science, University of Malaya, 50603 Kuala Lumpur, Malaysia; 3 Ecological Chemistry Group, Department of Chemistry, School of Chemical Science and Engineering, KTH, Royal Institute of Technology, Stockholm, Sweden; 4 Department of Chemistry, Faculty of Science, El-Menoufia University, Shebin El Kom, Egypt; 5 Department of Chemistry, COMSATS Institute of Information Technology, Abbottabad, 22060 Pakistan; 6 Agricultural Research Centre, Cairo, Egypt; 7 Department of Zoology, Faculty of Science, El-Menoufia University, Shebin El-Kom, 32512 Egypt; 8 Department of Molecular Biosciences, The Wenner-Gren Institute, Stockholm University, Stockholm, 106 91 Sweden; 9 Quaid-i-Azam University, Islamabad, 45320 Pakistan; 10 Pharmacognosy Department, College of Pharmacy, Cairo University, Kasr el Aini St., P.B. 11562, Cairo, Egypt; 11 Department of Pharmacognosy, College of Pharmacy, King Saud University, P.O. Box 2457, Riyadh, 11451 Saudi Arabia

**Keywords:** *Ixodes ricinus*, Essential oil, Chemical composition, Tick repellents, Gas chromatography-mass spectrometry, Egyptian flora

## Abstract

Due to the role of *Ixodes ricinus* (L.) (Acari: Ixodidae) in the transmission of many serious pathogens, personal protection against bites of this tick is essential. In the present study the essential oils from 11 aromatic Egyptian plants were isolated and their repellent activity against *I*. *ricinus* nymphs was evaluated Three oils (i.e. *Conyza dioscoridis* L., *Artemisia herba-alba* Asso and *Calendula officinalis* L.) elicited high repellent activity in vitro of 94, 84.2 and 82%, respectively. The most active essential oil (*C*. *dioscoridis*) was applied in the field at a concentration of 6.5 µg/cm^2^ and elicited a significant repellent activity against *I*. *ricinus* nymphs by 61.1%. The most repellent plants *C*. *dioscoridis*, *C*. *officinalis* and *A*. *herba-alba* yielded essential oils by 0.17, 0.11 and 0.14%, respectively. These oils were further investigated using gas chromatography-mass spectrometry analysis. α-Cadinol (10.7%) and hexadecanoic acid (10.5%) were the major components of *C*. *dioscoridis* whereas in *C*. *officinalis*, α-cadinol (21.2%) and carvone (18.2%) were major components. *Artemisia* *herba-alba* contained piperitone (26.5%), ethyl cinnamate (9.5%), camphor (7.7%) and hexadecanoic acid (6.9%). Essential oils of these three plants have a potential to be used for personal protection against tick bites.

## Introduction

Ticks belong to a group of exclusively blood-feeding ectoparasites (Elmhalli et al. 2009). From the medical point of view, ticks are the second most important group of disease vectors after mosquitoes. The pathogenic agents transmitted by ticks affect the public health and cause economic losses in livestock sector (Svehlova et al. 2014). The Mediterranean region offers suitable environment for a wide range of tick species (Maia et al. 2014). The geographical distribution of ticks has continued to increase over the past three decades (Tabanca et al. 2013; Svehlova et al. 2014). This distribution might be modified by future climate and environmental changes (Jore et al. 2014).

The common tick, *Ixodes ricinus* (L.) (Acari: Ixodidae), is a triphasic tick that parasitizes a large number of vertebrates including small, medium to large mammals; birds and lizards (Becker et al. 2009). The abundance of *I*. *ricinus* on different vegetation types in a wooded area of Southern Italy was determined by Dantas-Torres and Otranto (2013). They found that the abundance of *I*. *ricinus* larvae on the ground-level vegetation was generally higher than on the higher vegetation whereas both nymphs and adult stages were more abundant on higher vegetation. The questing behavior of *I*. *ricinus* consists of climbing the low vegetation to a point from where it can attach to the passing hosts. The host-seeking activity of the castor-bean tick indicated that this tick has a bimodal seasonal activity with a dominant peak during spring whereas a minor peak during autumn (Schulz et al. 2014). The immature stages (larvae and nymphs) feed on woodland birds and small to medium-sized mammals while the adult female feeds on large mammals. Each life stage (larva, nymph, adult) of this tick feeds once on different host for continuous period lasting several days (Heylen et al. 2013).

*Ixodes* *ricinus* is a potential vector of different pathogens. The cattle parasite, *Babesia divergens* (the causative agent of human’s babesiosis) is transmitted to human by *I*. *ricinus* (Zintl et al. 2014). Spirochete, *Borrelia* *burgdorferi* sensu lato (the causative agent of Lyme disease) is considered diderm (double-membrane) bacteria with a worldwide distribution. *Borrelia burgdorferi* is mostly associated with *I*. *ricinus* complex in Northern Hemisphere. The density of questing *I. ricinus* in Northern Norway was determined for the first time by Hvidstena et al. (2015). They found that the overall prevalence of nymphs and adult ticks infected by *B. burgdorferi* s.l. was 21 and 46%, respectively. The high incidence rate of reported Lyme borreliosis in Bonnoy region can be explained by the high *Borrelia*-infection prevalence in ticks (Hvidstena et al. 2015). The encephalitis virus is transmitted to humans by *I*. *ricinus* nymphs and adults. The infection prevalence of tick-born-encephalitis virus (TBE) was significantly lower in *I*. *ricinus* nymphs (0.51%) than adults (4.48%) stage (Pettersson et al. 2014). Due to the role of *I*. *ricinus* tick in the transmitting of many serous pathogens, personal protection against bites of the infected stages of *I*. *ricinus* is essential.

Despite recent advances in tick control strategies, large-scale reduction of tick populations has not been achieved (Tabanca et al. 2013). The synthetic repellents are commonly accepted means of personal protection against tick bites (Iori et al. 2005). However, the use of such commercial synthetic acaricides leads to resistance, residual effects and potentially can harm the environment (Elmhalli et al. 2009). There is a direct need to establish alternative substances for tick control which are safer, available, cheaper and more effective (Frances and Wirtz 2005). Many essential oils (extracted from medicinal plants) considered as promising repellent agents against *I*. *ricinus*. The repellency of the oils appears to be largely associated with the presence of volatile terpenoid constituents.

As a part of our ongoing studies of bioactive constituents from plants commonly used in folk medicine (El-Seedi et al. 2012; Al-Henhen et al. 2014; Boldbaatar et al. 2014 **)** with potential use in chemical ecology research, we here present results from 11 medicinal and culinary plants originating from Egypt. We isolated the essential oils and evaluated their bioactivity against the common tick *I*. *ricinus* and identified the main chemical constituents of these essential oils using gas chromatography-mass spectrometry (GC–MS).

## Materials and methods

### Essential oils

The air-dried ground parts of the plants (Table 1) were purchased from a commercial source in Cairo-Egypt. One hundred gram of each plant material was subjected to steam distillation for 4 h. The distillate was collected and extracted three times with 100 ml of HPLC grade n-hexane (VWR Int. Sweden), dehydrated using anhydrous magnesium sulfate (Alfa Aesar UK), filtered and solvent was evaporated using rotary evaporator at 20 °C under reduced pressure. The essential oils were weighed and reconstituted in hexane as 20 mg/ml, stored in tightly closed glass vials in freezer at −20 °C until further investigations.

**Table 1:** Essential oil yield of the 11 medicinal plants used in the current study

No.	Latin name	Common Egyptian name	Family	Part used	% yield (w/w)
1	*Ammi majus* L.	Khella barry	Apiaceae	Seeds	0.09
2	*Ammi visnaga* L.	Khella balady	Apiaceae	Seeds	0.08
3	*Foeniculum vulgare* Mill.	Shammar	Apiaceae	Seeds	1.1
4	*Nerium oleander* L.	Daflla	Apocynaceae	Leaf	0.07
5	*Artemisia herba-alba* Asso	Sheih balady	Asteraceae	Leaf	0.11
6	*Calendula officinalis* L.	Kanedula	Asteraceae	Flower	0.14
7	*Conyza dioscoridis* L.	Baranof	Asteraceae	Leaf	0.17
8	*Matricaria recutita* L.	Sheih baboning	Asteraceae	Flower	0.2
9	*Ricinus communis* L.	Kharwae	Euphorbiaceae	Seeds	0.01
10	*Lawsonia inermis* L.	Henna	Lythraceae	Leaf	0.15
11	*Lantana camara* L.	Lantana	Verbenaceae	Leaf	0.18

### GC–MS analysis

Separation and identification of volatiles from essential oils were carried out by GC–MS using a Varian 3400 GC connected to a Finnigan SSQ 7000 quadrupole mass spectrometer. 1 µl aliquot containing 3 µg/µl of essential oil was injected to GC injector for analysis. The GC was equipped with a split/split less injector (split less mode, 30 s; injector temperature, 230 °C; carrier gas, Helium with a constant pressure of 10 psi). A DB-WAX capillary column (30 m, 0.25 mm ID, and 0.25 µm film thickness, J & W USA) was used. The temperature program was: 40 °C for 1 min then increased with a rate of 4 °C/min up till 235 °C and held at 235 °C for 10 min. Transfer line connecting GC to the MS was isothermally set to 240 °C throughout the analysis. The temperature of MS ion source was 150 °C and mass spectra were obtained at 70 eV with a mass range of 30–400 *m/z*. Mass spectra of separated compounds were compared to the Finnigan NIST-2008 (National Institute of Standard and Technology) MS library and to available reference compounds (El-Seedi et al. 2008, 2010).

### Tick collection and maintenance

Nymphs of *I*. *ricinus* were collected from the field during summer 2009 in Stockholm, Sweden using methods described by Garboui et al. (2007) and El-Seedi et al. (2012). Briefly, two wooden poles of 1 m length and 3 cm diameter were attached to two opposite sides of a white flannel cloth (1 × 1 m) and a long string was tied on both ends of one wooden pool attached to the cloth. The cloth was dragged over the vegetation by holding string in hand whereas the two wooden poles made it possible to expand the cloth during dragging so that maximum cloth surface could be exposed and touch the vegetation. Nymphs attached to the cloth were removed with the help of soft forceps and put into tubes having wet filter paper. Nymphs were maintained in complete darkness at 4 °C and 80–95% relative humidity until they were used in laboratory bioassay. Before starting experiment the ticks were kept in room temperature for 24 h.

### Laboratory bioassay

To ascertain the repellent activity of the essential oils (EO) (at a concentration of 1 mg/ml) extracted from plants (Table 2) against *I*. *ricinus* nymphs, the method described by Jaenson et al. (2003) and Garboui et al. (2007) was used. The walls of transparent-plastic Falcon tubes (50 ml centrifugal tube: 116 × 29 mm) were perforated, to prevent the internal air saturation with the observer odor, test or control substances. A 100 µl of each EO solution (1 mg/ml) was applied evenly on a cotton cloth with a pipette to get a final concentration of 15 µg/cm^2^. Control cotton cloths were treated with 100 µl hexane. The treated and control cloths were air dried for 2 min for solvent evaporation. Freshly collected unfed *I*. *ricinus* nymph was introduced into Falcon tube and firstly tested with hexane treated cloth (that fixed to the Falcon upper end with the help of rubber band) for 5 min and immediately after the same nymph was tested against the EO treated cloth for another 5 min (one cloth used for each nymph). In order to simulate the nymph, the observer held his hand palm on the outer surface of the cloth during the observation period (5 min). The same observer conducted the entire bioassay who was attractive to ticks. A tick nymph was considered “attracted” to the cloth if it detached all its legs from the wall of the Falcon tube and clung towards the treated cloth within 5 min, whereas the nymph which failed to reach the cloth in this time or/and turned around the Falcon tube wall was regarded as “repelled” (El-Seedi et al. 2012). Ten nymphs were used/each oil/each replicate. Five replicates were used, thus the overall tick number was 50 nymphs for each EO and control group. The percentage of repellency was calculated using the formula of Jaenson et al. (2005) as follows:

**Table 2:** Percentage of repellency of essential oils of different plants based on percentages of *Ixodes ricinus* nymphs attracted to test and control (hexane) in lab bioassay

Tested essential oil	N	% attracted ticks		*P*	% repellency
		Control	Test		
*Ammi majus*	6	95	30	0.024	68.3
*Ammi visnaga*	6	80	30	0.026	62.4
*Foeniculum vulgare*	6	85	25	0.024	70.6
*Nerium oleander*	6	95	37	0.026	60.0
*Artemisia herba-alba*	6	95	15	0.024	84.2
*Calendula officinalis*	6	85	15	0.024	82.0
*Conyza dioscoridis*	6	83	5	0.02	94.0
*Matricaria recutita*	6	95	57	0.02	40.0
*Ricinus communis*	6	93	35	0.027	61.2
*Lawsonia inermis*	6	85	35	0.024	58.3
*Lantana camara*	6	95	35	0.014	63.3
98% DEET^a^	10	70	0	<0.001	100.0
19% DEET	10	84	4	<0.001	95.2
10% DEET	10	92	14	<0.001	84.8

^a^ *N*,*N*-diethyl-*m*-toluamide (DEET) data was extracted from Jaenson et al. (2003)

% repellency = [(number of nymphs recorded as “attracted” in the control vial − number of nymphs recorded as “attracted in the test vial)/number of nymphs recorded as “attracted” in the control vial] × 100.

### Field experiment

In order to test the tick repelling activity under natural conditions, a field trial was conducted using the similar white flannel cloths that used for tick collection mentioned above. The field trail was employed using method described by Garboui et al. (2007). Briefly, two persons dragged two white flannel clothes (1 × 1 m) on vegetation in parallel manner. One cloth was sprayed with 65 mg of the test substance dissolved in 100 ml hexane to cover the whole surface of the cloth whereas the other cloth was sprayed with 100 ml hexane control. The final concentration of essential oil was 6.5 µg/cm^2^ of the cloth. During the trial the clothes were dragged against the vegetation in such a way that the treated side was towards the ground vegetation to maximize the effect of the treatment. The cloths were slowly dragged over an area of 10 m^2^ before they were inspected and attached ticks were counted, removed and put into separate labeled vials. This procedure was repeated 20 times per day for the test substance and the control thus overall a tested cloth was dragged over a vegetation of 200 m^2^. The treated clothes were tested for two consecutive days and were stored separately in airtight plastic bags until the next day of testing. Temperature and humidity were recorded the day before, during testing days and one day after the test. The repellency of essential oil was calculated (Jaenson et al. 2005) using the following formula:

% repellency = [(no. of nymphs on control cloth – no. of nymphs on test cloth)/no. of nymphs on control cloth] × 100.

### Statistical analysis

Wilcoxon match pair test was employed on lab bioassay data whereas Mann–Whitney *U*-test was used to find the difference (α = 0.05) between control and essential oil treated clothes. The tests were performed in SPSS 20.0 (IBM USA).

## Results

### Isolation of essential oils

Eleven plants originating from Egypt were investigated as potentially active against blood sucking ticks. The Egyptian plants used in the present study (Table 1) were found to be rich in essential oils, which were obtained by steam distillation. The largest yield of essential oil was obtained from the seeds of *Foeniculum vulgare* Mill. (1.1%) followed by *Matricaria recutita* L. (0.2%), whereas *Ricinus communis* L. exhibited the lowest yield (0.01%). Furthermore, the oils obtained from *Conyza dioscoridis* L., *Calendula officinalis* L. and *Artemisia herba-alba* Asso were 0.17, 0.14 and 0.11% yield, respectively (Table 1).

### Chemical analysis

The constituents of each plant essential oil and their relative percentage based on the total ion current chromatogram are summarized in Table 3. Most of the compounds were oxygented mono- and sesquiterpenoids and a few aromatic monoterpenes. In the essential oils the number of compounds, representing percentage of oil, were identified as follows; *Ammi majus* 20 (87.7%), *A*. *visnaga* 13 (95.3%), *F. vulgare* 6 (98.1%), *Nerium oleander* 33 (93.5%), *A. herba alba* 21 (91.3%), *C. officinalis* 24 (85.4%), *C. dioscoridis* 33 (84.1%), *M. recutita* 12 (94.1%), *R. communis* 26 (86.4%), *Lawsonia inermis* 27 (67.2%), *Lantana camara* 15 (80.9%), are presented in Table 3.

**Table 3:** Identified compounds in the essential oils of the plants used in the present study

Volatile name	*Ammi majus*	*Ammi visnaga*	*Foeniculum vulgare*	*Nerium oleander*	*Artemisia herba-alba*	*Calendula officinalis*	*Conyza dioscoridis*	*Matricaria recutita*	*Ricinus communis*	*Lawsonia inermis*	*Lantana camara*
*Terpenes hydrocarbons*											
Cadinene						9.1	2.3		1.9		0.8
β-Caryophyllene											32.5
α-Humulene											12.5
β-Cubebene											4.2
δ-Elemene											10.6
β-Farnesene				0.4				5.4			
τ-Gurjunene											2.7
Limonene		0.9	5.2								
α-Muurolene						1.2					
Total		0.9	5.2	0.4		10.3	2.3	5.4	1.9		63.3
*Oxygenated monoterpenes*											
Borneol					2.2						
Camphor					7.7						0.8
3-Carene, 10-(acetylmethyl)				3.1							
Carvacrol				1.8	0.7						
Carvone	4.2	57	0.2	13.2		18.2	3.7	3.3	9.9		
Carveol		1.1									
1,8-Cineol											3.3
Dihydrocarvone	7	2									
Dihydrocarvoyl acetate									1.4		
Fenchone			3.9								
Geranyl acetone				1.6							
2-Hydroxypiperitone					1						
β-Ionone						1.1					
α-Isophoron					2.1						
Linalool	1.5			0.6		0.4	1.3		1.1	1.1	
4-Methylisopulegone					3.5						
4-Oxoisophorone					1.2						
Piperitone	3.6			0.9	26.5						
Pulegone				3.4		3.8	0.9	0.6	3.8		
4-Terpineol				1.7		2.6	0.7		2.6		0.6
α-Terpineol				1.1		0.7	0.7		0.9		
Thymol				2.1	2.3						
Yomogi alcohol							2.4	0.6			
Total	16	60.1	4.1	29.5	47.2	26.8	9.7	4.5	19.7	1.1	4.7
*Oxygenated sesquiterpenes*											
Bisabolol oxide A								67			
α-Bisabolol								0.8			
α-Bisabolol oxide B								8.8			
α-Cadinol						21.2	10.7				
δ-Cadinol						1.3	1.2	2			
τ-Cadinol						3.7	4.7				
β-Caryophyllene oxide							0.5				2.5
Cubenol						1.5	1				
Diepi-α-cedrene epoxide							1.6				
α-Eudesmol							2.1				
β-Eudesmol							3.4				
4,4-Dimethyltetracyclo-(6,3,2,0) (2,5)0(1,8)tridecan-9-ol							6.1				
Guaiol						3.8					
Hexahydrofarnesyl acetone	0.9			2.4	5.7		2.5			1	
τ-Muurolol						5.3	3.5				
Neoisolongifolene-8-ol											1.5
Nerolidol											4
Spathulenol							0.6	1.3			2.4
Viridiflorol						1.4					
Total	0.9			2.4	5.7	38.2	37.9	79.9		1	10.4
*Aromatics*											
2-Acetylfuran										4.8	
*p*-Allylanisole			88	0.7		0.5			1.5		
Apiol-1	3.8	18.1									
Apiol-2		3.6									
β-Asarone				10							
Benzaldehyde						0.4	2				
Benzyl alcohol					2.4				0.9	1.1	
Coumaran				0.8	1.4				24.7	1.4	
Dihydroactinidiolide				2.6	1.1	1.7	2.1				
2,4-Di-tert-butylphenol									1.7		
Eugenol	1.2			2.7						1.8	
Ethyl cinnamate					9.5						
4-Ethylphenol										1.5	
Furfural						0.7	1.2			20.8	
Furfuryl alcohol							0.8			4.5	
4-Hydroxybenzaldehyde									4.9		
2-Methoxyphenol										1.9	
2-Methoxy-4-vinylphenol	0.9	1.2		1.1	2.9					1.3	
Methyl cinnamate				1					1.4		
2-Methyl-3-phenyl-propanal				1.8							
4-Methylphenol										1	
4-Methylphenylethanol					5						
4-Methoxycarbonylimidazole										0.8	
5-Methyl-2-furfural										14.5	
Myristicin		1.7									
5-Pentylresorcinol				0.6						1	
Phenol										2.6	
Phenylethanol				6.5					2.6		
*p*-Propenylanisole	0.7		0.7	2.6		2.8		1.8	4.3		
1-(2,3,6-Trimethylphenyl)-3- buten-2-one										1.5	
Visnagin		2.2									
Total	6.6	26.8	88.7	30.4	22.3	5.4	4.9	1.8	42	34.9	
*Aliphatic carboxlic fatty acids*											
Decanoic acid	0.9			0.3	2			1.9			
Dodecanoic acid	2.8				2.2	0.9					
Hexadecanoic acid	38	4	0.1	16.9	6.9	2.2	10.5		7.9	6.4	0.9
Hexanoic acid		0.8		2.5			4.6	0.6		1.6	
2-Hexenoic acid		1.8					1				
5-Hexenoic acid							1.4				
Linolic acid	3.5								1.8		
3-Methylbutanoic acid							2.3				
3-Methylpentanoic acid							1.7				
4-Methyl-2-pentenoic acid							2				
Nonanoic acid	2.3			1.4			1.1				
9-Octadecenoic acid									1		
6-Octadecenoic acid	6.4								0.8		
Octanoic Acid	1.3	0.9		1.2						0.7	
Tetradecanoic acid	4.9				2.7	1.6	2			3.9	
Undecanoic acid	1.1										
Total	61	7.5	0.1	22.3	13.8	4.7	26.6	2.5	11.5	12.6	0.9
*Aliphatic esters*											
Ethyl butyrate				1.7							
1-Hydroxy-2-butanone acetate										2.8	
3-Hydroxy-2,4,4-trimethyl pentyl 2-methylpropanoate				3.3							
Methyl hexadecanoate				1.8					0.9		
Methyl stearate									1.8		
Methyl oleate									3.3		
10,13-Octadecadienoic acid, methyl ester									1.3		
Vinyl propionate										3.1	
Total				6.8					7.3	5.9	
*Aliphatic alcohols and ketones*											
Acetoxypropanone										5.1	
9-Hexadecen-1-ol	1.5										
3-Hexen-1-ol										1.7	
6-Methyl-5-heptene-2-one						1.7					
9-Methyl-5-methylene-8-decen-2-one	1.2										
Nona-3,5-dien-2-ol					2.3						
Nonanol									2.7		
Octanol									1.3		
Phytol				1.7			2.7				1.6
3,7,11,15-Tetramethyl-2- hexadecen-1-ol										4.9	
Total	2.7			1.7	2.3	0	2.7		4	11.7	1.6
Total no. of identified compounds	20	13	6	33	21	24	33	12	26	27	15
Total % of identified compounds	87.7	95.3	98.1	93.5	91.3	85.4	84.1	94.1	86.4	67.2	80.9

The values show the relative chemical composition of essential oil

Compounds present in each group are listed according to their eluting order on a DB-Wax capillary column

Major components of *A*. *majus* essential oil were hexadecanoic acid (38%), dihydrocarvone (7%), 6-octadecenoic acid (6.4%) and tetradecanoic acid (4.9%) representing 56.3% of oil. The main compounds of *A*. *visnaga* essential oil were carvone (57%) and apiol (18.1%) constituting 75.1% of oil (Table 3).

The *A*. *herba alba* essential oil contained piperitone (26.5%), ethyl cinnamate (9.5%), camphor (7.7%) and hexadecanoic acid (6.9%) as major compounds representing 50.6% of the oil. The major constituents of *C*. *officinalis* oil were α-cadinol (21.2%), carvone (18.2%) and cadinene (9.1%) those representing 48.5% of the oil (Table 3).

The main compounds of *C*. *dioscoridis* oil were α-cadinol (10.7%), hexadecanoic acid (10.5%), 4,4-dimethyltetracyclo-(6,3,2,0)(2,5)0(1,8)tridecan-9-ol (6.1%), τ-cadinol (4.7%), hexanoic acid (4.6%), carvone (3.7%) and τ-muurolol (3.5%) and representing 43.8% of the oil (Table 3). *p*-Allylanisole (88%) and limonene (5.2%) constituted the major part of *F. vulgare* essential oil.

β-Caryophyllene (32.5%), furfural (20.8%), bisabolol oxide A (67%), hexadecanoic acid (16.9%) and coumaran (24.7%) were the major compounds of *L*. *camara*, *L*. *inermis*, *M*. *recutita*, *N*. *oleander* and *R*. *communis* essential oils, respectively (Table 3).

### Laboratory bioassay

Experimental results revealed that all essential oils from eleven Egyptian plants were to various degree repellent to *I*. *ricinus* nymphs. The most active essential oils were *C*. *dioscoridis*, *A*. *herba alba* and *C*. *officinalis* that elicited strong repellent activity against tick nymphs by 94, 84.2 and 82.0%, respectively (Table 2). The *F. vulgare* and *A. majus* oils revealed moderate repellent activity by 70.6 and 68.3%, respectively. The oils from *L*. *camara*, *A. visnaga*, *R*. *communis*, and *N*. *oleander* elicited repellency by 63.3, 62.4, 61.2, and 60%, respectively. A minimum repellent activity was observed by the oils of *L*. *inermis* and *M*. *recutita* (Table 2).

### Field study

**Fig. 1:**
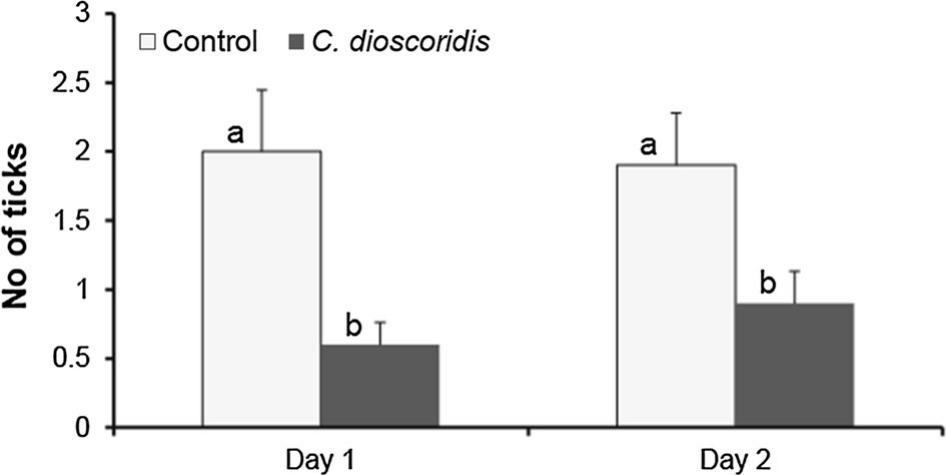
Mean number of ticks attracted toward control and *Conyza dioscoridis* essential oil treated clothes in the field trial. Columns with different letters are significantly different from each other (*P* < 0.05)

Laboratory bioassay data revealed that the essential oil of *C. dioscoridis* exhibited strongest repellency toward ticks compared to all other essential oils. Therefore, the essential oil of *C. dioscoridis* was applied in the field trail at a concentration of 6.5 µg/cm^2^. This oil elicited a significant repellent activity against *I*. *ricinus* nymphs on two consecutive days (*P* < 0.05, Fig. 1).

## Discussion

There is a great potential for the use of plants from Africa, Asia and South America tropical and subtropical regions for finding new bioactive molecules and for our purposes new compounds for tick control (Habeeb 2010). In Table 4 numerous candidates with putative repellency activity are listed and oxygenated monoterpenes and sesquiterpenes are among the most frequently identified compounds. A number of plants have earlier shown repellent activity against *I. ricinus* nymph and the majority of the compounds are oxygenated monoterpenes (Table 4). The present study exhibited repellent activity of essential oils extracted from eleven Egyptian medicinal plants against *I*. *ricinus* nymphs. Among the studied essential oils *C*. *dioscoridis*, *A*. *herba alba* and *C*. *officinalis* exhibited a strong repellency for ticks in laboratory bioassays. These three plants are belonged to Asteraceae family and showed the presence of a number of major compounds that might be responsible for their bioactivity.

**Table 4:** Plants with repellent activity against *Ixodes ricinus* and some of their major chemical constituents which might contribute to their activity

Plant essential oil	Family	Plant part	Chemical ingredients	% repellency	References
*Cymbopogon* spp. (10% (w/w) in ethanol)	Poaceae	Commercial oil (Stockholms Aether & Essence fabrik)	Citronellol, Geraniol	90 after 6 h	Thorsell et al. (2006)
*Syzygium aromaticum* (10% (w/w) in ethanol)	Myrtaceae	Commercial oil (Stockholms Aether & Essence fabrik	Eugenol	82 after 6 h	Thorsell et al. (2006)
*Rhododendron tomentosum* (10% in acetone)	Ericaceae	Leaves	Palustrol (22.8%), Myrcene (21.3%), Ledol (6.1%), 2,6-Dimethyl-1,5,7-octatriene-3-ol (4.1%), Alloaromadendrene (2.8%)	95.1 (laboratory experiment)	Jaenson et al. (2005)
*Myrica gale* (10% in acetone)	Myricaceae	Leaves	(3Z)-Hexenol (18.3%), 4-Hydroxy-4-methylpentane-2-one (10.2%), Cadinadiene (8.4%), α-Trpineol (6.2%), 4-Terpineol (5.8%)	48.5 (laboratory experiment)	Jaenson et al. (2005)
*Corymbia citriodora* (30% in acetone for field experiment)	Myrtaceae		*cis*- and *trans*- *p*-Menthane-3,8-diol (PMD)	100 (laboratory experiment), 85 (field experiment)	Jaenson et al. (2006)
*Pelargonium graveolens* (30% in 1,2-propanediol)	Geraniaceae		No chemical composition reported	100 (laboratory experiment)	Jaenson et al. (2006)
*Lavandula angustifolia* (30% in 1,2-propanediol)	Lamiaceae		No chemical composition reported	100 (laboratory experiment)	Jaenson et al. (2006)
*Mentha spicata* (0.1% w/v in hexane) (15% µg/cm^2^ for lab experiment, 6.5 µg/cm^2^ for field experiment)	Lamiaceae	Leaves	Carvone (54.7%), Pulegone (14.2%), 1,8-Cineole (4.7%)	93.2 (laboratory experiment), 59.4 (field experiment)	El-Seedi et al. (2012)
*Ocimum basilicum* (0.1% w/v in hexane) (15% µg/cm^2^)	Lamiaceae	Leaves	Linalool (27.8%), Estragole (12.3%), Methyl *trans* cinnamate (11.8%), Eugenol (9%), 1,8-Cineole (6.6%)	64.5 (laboratory experiment)	El-Seedi et al. (2012)
*Rosmarinus officinalis* (0.1% w/v in hexane) (15% µg/cm^2^ for lab experiment, 6.5 µg/cm^2^ for field experiment)	Lamiaceae	Leaves	1,8-Cineole (51.8%), Borneol (17.5%), Camphor (12.8%)	100 (laboratory experiment), 68.3 (field experiment)	El-Seedi et al. (2012)
*Origanum majorana* (0.1% W/V in hexane) (15% µg/cm^2^)	Lamiaceae	Leaves	4-Terpineol (55.6%), α-Terpineol (9.5%), Linalool (3.7%)	84.3 (laboratory experiment)	El-Seedi et al. (2012)

Usually the biological activity of a plant extract is due the presence of one or more major compounds (El-Seedi et al. 2012) but in the case of *C*. *dioscoridis* essential oil, it seems the greater activity is due to presence of blend of diverse type of compounds which might have additional or synergistic effects. However, in case of *C*. *officinalis* there was a number of major compounds which could be responsible for its higher activity. *C*. *dioscoridis* and *C*. *officinalis* produced the same oxygenated sesquiterpene as major compounds but the amount and bioactivity was slightly different between species. The most abundant compound of these plant oils was α-cadinol that was detected only in the essential oils of these two plants. α-Cadinol was found to be highly effective for controlling two house mite species *Dermatophagoides pteronyssinus* and *D. farinae* (Chang et al. 2001).

The chemical composition of *C*. *dioscoridis* in the present study is partly in accordance with Grace (2002), where he presented the essential oil of *Pluchea* (=*Conyza*) *dioscoridis* which consisted of both sesquiterpene hydrocarbons and oxygenated sesquiterpenes in large proportions with α-cadinol as the major constituent. However, our results are different from other previous studies (Nassar et al. 2014; Elshamy et al. 2015). Both studies showed that the major constituents of *C. dioscoridis* essential oil were mainly sesquiterpene hydrocarbons comprising more than 40% of the oil whereas there are more of oxygenated sesquiterpenes in the present essential oil. This difference in chemical composition might partly be explained by harvesting time, soil fertility, cultivation and drying methods of the plant materials have a significant impact on the chemical composition of essential oils (Okoh et al. 2007, 2008; Hussain et al. 2008; Omer et al. 2008; Antal et al. 2011). The effect of soil type and the seasonal variations on the quality and quantity of essential oil constituents was investigated by Omer et al. (2008) and Hussain et al. (2008). There is also a correlation between the age of plants and their essential oil yield and composition (Okoh et al. 2007) as well as harvesting season of three rose-scented geranium (*Pelargonium grayeolens* L’Her ex Ait; Geraniaceae) cultivars (Verma et al. 2013).

The Egyptian *C*. *officinalis* studied here with α-cadinol as major component of the essential oil and is in accordance with previous studies conducted in South Africa (Okoh et al. 2007) and Brazil (Gazim et al. 2008). The effect of plant age on the yield and constituents of the oil extracted from the *C*. *officinalis* grown in South Africa was determined by Okoh et al. (2007) and found that the most interesting stage is the post-flowering period, the oil of which rich in α-cadinene, α-cadinol, τ-muurolol, limonene and 1,8-cineole. Gazim et al. (2008) stated the presence of α-cadinol in Brazilian *C. officinalis* essential oil. The species has a long history in traditional medicine as Ibn El Bitar reported the use of this species in the treatment of epilepsy and as remedy for cold, colic and rheumatic pains (Boulos and El-Hadidi 1984).

Furthermore, different chemotypes of *A*. *herba alba* have been previously described originating from different localities. Identification of Sinai chemotype was achieved by GC–MS and two main oils were discerned, the cineole-bornane type and pinene type. The oils were rich in monoterpenes but did not contain any sesquiterpene components (Feuerstein et al. 1986). This is not consistent with our findings where we found piperitone as main compound. Essential oil of *A. herba alba* from Israel revealed the presence of sesquiterpene lactone and oxygenated monoterpenes (Segal et al. 1985). The population in Israel consist of a larger number of chemotypes of *A*. *herba alba* than was previously believed (Fleisher et al. 2002). An additional *A*. *herba alba* chemotype was described in Spain (Salido et al. 2004). Some Tunisian *A*. *herba alba* chemotypes showed the presence of similar compounds as in our essential oil however, the composition of the constituents was different (Mohsen and Ali 2009). Another study from *A. herba alba* essential oil from Tunisia described the presence of a number of potential tick repellent compounds; α-thujone (24.9%), germacrene D (14.5%), camphor (10.8%), 1,8-cineole (8.9%), β-thujone (8.3%), chrysanthenone (4.7%) and borneol (3.1%) in Kadri et al. (2011).

The repellency of *A*. *majus* (68.3%) essential oil towards tick *I*. *ricinus* in the present study could be explained by the presence of carvone, dihydrocarvone and piperitone. These three in combination are possible candidates for the repellent activity. The essential oil of *F*. *vulgare* consisted of only six compounds contributing more than 98% of the oil. Limonene, *p*-allylanisole, and fenchone were the most abundant compounds in this essential oil that could be responsible for its biological activity. Limonene is used as an insecticide to control ectoparasites and has activity against many plant-feeding insects as the pine weevil (Nordlander 1990), mites and microorganisms (Ibrahim et al. 2001). Fenchone is reported to show mosquito repellent activity (Kim et al. 2002).

*Ricinus* *communis*, *A*. *visnaga* and *N*. *oleander* showed a moderate activity which might partly be due to the presence of carvone. Previously, carvone has shown antifeedants properties for the pine weevil *Hylobius abietis* (Schlyter et al. 2004) and repellent activity against flour beetle *Tribolium castaneum* (Caballero-Gallardo et al. 2011).  The Environmental Protection Agency (EPA, USA, 2009) was reviewing a request to register it as a pesticide. *S*-(+)-Carvone is also used to prevent premature sprouting of potatoes during storage, being marketed in the Netherlands for this purpose under the name Talent (de Carvalho and da Fonseca 2006).

All the analyzed plants, exhibited repellent behavior against *I*. *ricinus* and all the oils contained oxygenated terpenes. However, the most active essential oils did not have similar volatile profiles. The current result strongly indicate that the oils contain a number of compounds having repellent properties and that many of them belong to the oxygenated group of monoterpenes and sesquiterpenes. The recent analyses did not include separation of the enantiomers of the chiral constituents. This lacking information might explain why essential oils with similar constituents have different repellent activity. However, it seems unlikely to find a specific compound in common that can explain the whole repellent activity, and most probably there is a combined effect of several constituents (Jaenson et al. 2005). The essential oils containing many tick repellent compounds might then be more useful and sustainable in tick control strategies.

Our study evaluated the potential of 11 plant essential oils and the oil of *C*. *dioscoridis* was found to be the most effective in the laboratory bioassay, moreover, it also proved to be a good repellent in the field trial. Therefore, *C. dioscoridis* oil might be useful sources of chemicals for controlling arthropods of medical, veterinary, or agricultural importance.
